# Nanopore sequencing enables novel detection of deuterium incorporation in DNA

**DOI:** 10.1016/j.csbj.2024.09.027

**Published:** 2024-10-03

**Authors:** Christian Höner zu Siederdissen, Jannes Spangenberg, Kevin Bisdorf, Sebastian Krautwurst, Akash Srivastava, Manja Marz, Martin Taubert

**Affiliations:** a RNA Bioinformatics and High-Throughput Analysis, Friedrich Schiller University Jena, Jena, Germany; b Aquatic Geomicrobiology, Institute of Biodiversity, Faculty of Biological Sciences, Friedrich Schiller University Jena, Dornburger Str. 159, 07743 Jena, Germany; c Balance of the Microverse, Cluster of Excellence, Friedrich-Schiller-University Jena, Grüne Aue, 07745 Jena, Germany; d European Virus Bioinformatics Center, Jena, Germany; e German Center for Integrative Biodiversity Research (iDiv) Halle-Jena-Leipzig, Leipzig, Germany; f FLI Leibniz Institute for Age Research, Jena, Germany

**Keywords:** Stable isotope probing, D_2_O labeling, ^13^C labeling, Deuterium, Oxford nanopore sequencing, Bayesian logistic regression

## Abstract

Identifying active microbes is crucial to understand their role in ecosystem functions. Metabolic labeling with heavy, non-radioactive isotopes, i.e., stable isotope probing (SIP), can track active microbes by detecting heavy isotope incorporation in biomolecules such as DNA. However, the detection of heavy isotope-labeled nucleotides directly during sequencing has, to date, not been achieved.

In this study, Oxford nanopore sequencing was utilized to detect heavy isotopes incorporation in DNA molecules. Two isotopes widely used in SIP experiments were employed to label a bacterial isolate: deuterium (D, as D_2_O) and carbon-13 (^13^C, as glucose). We hypothesize that labeled DNA is distinguishable from unlabeled DNA by changes in the nanopore signal. To verify this distinction, we employed a Bayesian classifier trained on signal distributions of short oligonucleotides (*k*-mers) from labeled and unlabeled sequencing reads.

Our results show a clear distinction between D-labeled and unlabeled reads, based on changes in median and median absolute deviation (MAD) of the nanopore signals for different *k*-mers. In contrast, ^13^C-labeled DNA cannot be distinguished from unlabeled DNA. For D, the model employed correctly predicted more than 85% of the reads. Even when metabolic labeling was conducted with only 30% D_2_O, 80% of the obtained reads were correctly classified with a 5% false discovery rate.

Our work demonstrates the feasibility of direct detection of deuterium incorporation in DNA molecules during Oxford nanopore sequencing. This finding represents a first step in establishing the combined use of nanopore sequencing and SIP for tracking active organisms in microbial ecology.

## Graphical abstract

**Figure:**
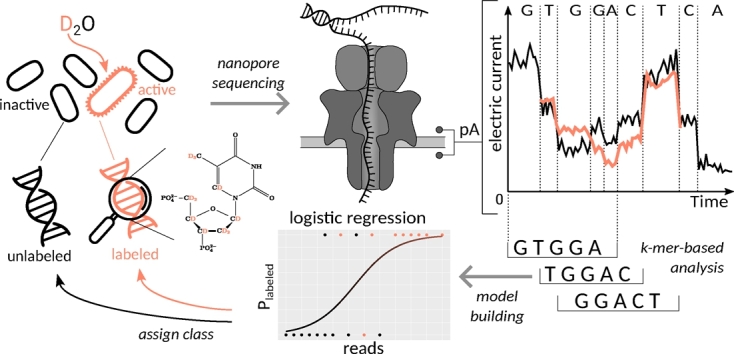

## 1 Introduction

Prokaryotic microbes (Bacteria and Archaea) play an indispensable role in the creation and maintenance of Earth's ecosystems by performing a variety of unique metabolic processes in global biogeochemical cycles. To understand the associated ecosystem functions, it is essential to identify which microbes are active in an ecosystem under specific conditions [29], [28]. The recent advances in high-throughput sequencing techniques, allowing the recovery of thousands of microbial genomes from environmental samples [4], [33], [2], have greatly facilitated the acquisition of information on the taxonomy and functional potential of microbial communities. While this information provides a basis for the construction of hypotheses regarding microbial activity, it does not sufficiently support verification of these hypotheses. To identify the active key players in microbial communities, other approaches are required.

In microbial ecology, metabolic labeling approaches with heavy, non-radioactive isotopes, so called stable isotope probing (SIP), have become a central method for tracking active microbes [29], [15]. In a SIP experiment, an isotopically labeled substrate of interest is added to the microbial community under investigation. Active microorganisms involved in the conversion of this substrate will then incorporate the heavy isotopes into their biomolecules. Various methods have been described for detecting heavy isotope incorporation in different classes of biomolecules, such as DNA [30], RNA [24], phospholipid-derived fatty acids [10], proteins [39], or even on the level of entire microbial cells [27], [6], [40]. Detection of heavy isotopes in biomolecules or cells is then regarded as evidence for the activity of the respective microbial taxon. The advantage of this method is that information on taxonomic identity and activity can be obtained from the same analytes. However, the current state-of-the-art method for DNA-SIP employs a complicated, two-step, wet-lab process [17]. First, DNA is manually fractionated through 48 to 72 hours of ultracentrifugation in a caesium chloride gradient to separate labeled, heavier, and unlabeled, lighter DNA based on differences in buoyant density. The DNA fractions obtained are subsequently sequenced to determine the distribution of individual taxa in the density gradient and compare this across samples to deduce labeling. This process is labor-intensive and error-prone, as contaminations and “carryover” DNA can be misinterpreted as coming from active organisms. It would be desirable, therefore, to be able to detect heavy isotope incorporation directly during sequencing.

**Fig. 1:**
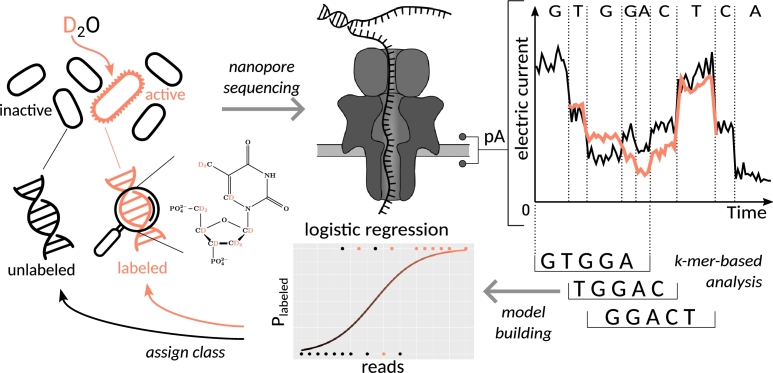
An overview of the isotope detection workflow for Oxford nanopore data is presented in this study. In the **left column**, deuterium (orange, applied using D_2_O at levels of 30% or 100% in this study) is incorporated into the genome of bacteria that are intended to be isotopically labeled. **Top center:** Bacterial DNA is sequenced using canonical Oxford nanopore sequencing workflow. **Top right:** Following the canonical Oxford nanopore workflow, basecalling is performed. Findings revealed that the inclusion of isotopes did not have a detrimental effect on basecalling quality or segmentation. **Bottom right:** K-mers are extracted from the basecalled data, along with normalized signal information and summary statistics for each *k*-mer from each read. **Bottom center:** A generalized linear model is trained to identify differences in signal median, signal median absolute deviation, dwell time median, and read statistics based on the presence or absence of D_2_O. **Bottom center/left:** The resulting model demonstrates high specificity and sensitivity in recognizing isotopically labeled reads.

The development of nanopore technology for DNA sequencing opened up new methods of detecting DNA modifications [21], [20], [26]. In nanopore sequencing, individual DNA strands are guided through a protein pore embedded in a membrane. Voltage is applied across the membrane, and the ionic current passing through the pore is measured. A DNA molecule passing through the pore restricts the flow of ions, and nucleotides currently occupying the pore determine the magnitude of this reduction. From the changes observed in the recorded current, it is possible to deduce the sequence of the DNA molecule as it moves through the pore. Moreover, modifications of the DNA, such as methylations, also lead to a change in the current, and can thus be detected [22]. The aim of this study was to test whether modifications to the DNA through the incorporation of heavy isotopes could also be detected using ONT nanopore sequencing (see Fig. 1). In contrast to classical nucleotide-specific approaches for detecting modifications, the goal was to establish a read-level based classification to distinguish between labeled and unlabeled DNA molecules. For this purpose, DNA from a bacterial isolate labeled separately with two heavy, non-radioactive isotopes, deuterium (^2^H resp. D) and carbon-13 (^13^C) was employed. These isotopes are commonly used in SIP experiments, with D being applied as D_2_O for labeling all active organisms in a community [6], [40], [16], and ^13^C being used to track the flux of carbon from specific carbon compounds [29], [38]. We hypothesize that either of these isotopes will have a detectable influence on the ionic current passing through the protein pore during nanopore sequencing, and hence, reads from labeled and unlabeled DNA can be distinguished. To achieve this, a Bayesian classifier was trained using the signal distributions of short oligonucleotides, the *k*-mers, from both the labeled and unlabeled sequencing reads. Additionally, false discovery rates were determined to allow assessment of the reliability of the established classification model. The ability to detect isotope labeling directly during sequencing has the potential to aid in the development of new SIP techniques in the future.

## 2 Methods

### 2.1 Generation of isotope-labeled DNA

For metabolic labeling with D_2_O and ^13^C, a bacterial isolate affiliated with the alphaproteobacterial genus *Aminobacter* was used. *Aminobacter* P9b was isolated from the groundwater of well H41 (51.197456 N 10.755476 E) of the Hainich Critical Zone Exploratory on R2Amod agar (Carl Roth GmbH + Co. KG, Karlsruhe, Germany). A pre-culture was grown in minimal medium (0.4 g/L K_2_HPO_4_, 0.8 g/L NH_4_Cl, 0.1 g/l MgSO_4_ × 7 H_2_O, 10 mM HEPES, 5 ml/l trace element solution “T” [8], pH 7.2) and 50 mM glucose. For labeling with D_2_O, the same minimal medium was prepared with pure D_2_O (Merck KGaA, Darmstadt, Germany). Medium with 30% (v/v) D_2_O was prepared by mixing 100% D_2_O and unlabeled medium. Parallel incubations of *Aminobacter* P9b in medium with 0%, 30% and 100% D_2_O and glucose were set up: Cells from 0.5 ml of the pre-culture were pelleted by centrifugation at 2800×g for 8 minutes, and washed once with the respective medium. Subsequently, cells were resuspended in 10 ml of the respective medium and transferred into 20 ml glass culture tubes. The tubes were sealed with butyl rubber stoppers to prevent the exchange of D_2_O with the atmosphere and incubated for 48 h at room temperature with shaking at 120 rpm. For labeling with ^13^C, parallel incubations with U-^13^C_6_-glucose (≥ 99 atom % ^13^C, Merck KGaA) and unlabeled glucose were conducted using the same setup and incubation conditions.

Following incubation, cells were harvested by centrifugation at 10,000×g for 10 minutes. Pellets were resuspended in 2.6 ml sucrose-EDTA-Tris buffer (0.75 M sucrose, 40 mM disodium-EDTA, 50 mM Tris base pH 9) and DNA was extracted using a phenol-based protocol, as previously described [38]. During extraction, pipetting and shaking were reduced to a minimum to retain long DNA molecules for nanopore sequencing. The concentration of the DNA extracted was quantified using a Qubit 3.0 fluorometer (Fisher Scientific GmbH, Schwerte, Germany) and the Qubit dsDNA broad-range assay kit (Fisher Scientific GmbH). DNA extracts were stored at 4 °C prior to library preparation for sequencing.

### 2.2 Nanopore sequencing

Library preparation was conducted with the five DNA extracts obtained from the metabolic labeling with D_2_O and ^13^C. Libraries for Oxford Nanopore Technologies (ONT) sequencing were prepared using the Native Barcoding amplicons protocol with kits EXP-NBD104, EXP-NBD114, and SQK-LSK109 (ONT, Oxford, United Kingdom). This was done following the manufacturer's instructions but with slight modifications: An input mass of 2−4 μg DNA was taken for each sample to ensure a high-yield library. For DNA end repair, the NEBNext Companion Module for Oxford Nanopore Technologies Ligation Sequencing (New England Biolabs GmbH, Frankfurt am Main, Germany) was used, and incubation times were increased from the recommended 5 minutes to 20 minutes. Stringent cleanup was performed with 0.66 volumes of AMPure XP beads (Beckman Coulter GmbH, Krefeld, Germany), followed by elution at 50 ^∘^C to enrich the libraries with high molecular weight DNA molecules. The incubation times for the barcode and adapter ligation were doubled to 20 minutes and the amount of Adapter Mix II (AMII) was reduced to 3.5 μl to improve ligation efficiency. For the D_2_O labeling experiment, the 0%, 30% and 100% D_2_O samples were pooled in equal ratios, to be sequenced on the same flowcell. For the ^13^C labeling experiment, the same was done for the unlabeled and ^13^C labeled samples. The concentration of the resulting two final libraries was adjusted to 400−500 ng of DNA in 12 μl water. The individual libraries were loaded on separate R9.4.1 flowcells and sequenced on a MinION MK1B device (ONT). The sequencing run was terminated following 72 h and 84 h, respectively, for D_2_O and ^13^C samples. The sequencing reports are attached as supplemental files. The Oxford Nanopore pod5 files can be found under https://dx.doi.org/10.5281/zenodo.11105094 for the 0%, 30%, 100% D_2_O reads and https://dx.doi.org/10.5281/zenodo.11105021 for ^12^C and ^13^C reads.

### 2.3 Basecalling and segmentation of sequencing data

**Basecalling:** Basecalling was performed using the Guppy basecaller v6.4.8 (ONT) with the dna_r9.4.1_450bps_sup model, which, according to ONT, has the highest accuracy. Quality-score filtering during basecalling was disabled by using the parameter disable_qscore_filtering, in case isotope incorporation would impair basecalling quality. The attached barcodes were used to assign reads to samples during basecalling using the parameter --barcode_kits EXP-NBD114, and were subsequently trimmed together with adapters using the parameters --trim_strategy dna and --enable_trim_barcodes.

**Assembly:** The reference sequences for *Aminobacter* P9b were assembled from the individual biological samples. For the 100% D_2_O and the ^12^C sample, random sub-sampling of reads was performed prior to assembly due to the high read number obtained in these samples (see Table 1). Draft assemblies for each sample were built with Flye v2.9 [18] using the additional parameters --scaffold to enable scaffolding and --nano-raw. Reads were mapped against the draft assembly with minimap2 v2.26 [19] for each sample, using the parameter -x map-ont. Subsequently, racon v1.5 [41] (with scoring parameters -m 8 -x -6 -g -8) and medaka v1.8 (ONT) (with the parameter -m ‘r941_min_sup_g507’ matching the basecalling model from Guppy) were used to polish the draft assemblies. To compare the assemblies, pairwise alignments were constructed with nucmer v3.1 and analyzed with dnadiff v1.3 [25]. The final assembly of the ^12^C sample is available at NCBI under BioProject accession PRJNA1089141.

**Table 1:** **Overview of sequencing quality and yield.** Shown are the number of sequenced reads and the number of basecalled bases, as well as medians and standard deviations of the PHRED score as a measure for read quality, for each individual sample. Note the tenfold higher number of bases for the 100% D_2_O sample as compared to the other D_2_O samples.

Sequencings	# reads	# bases	PHRED median	PHRED std. dev.
0% D_2_O	102,220	1.595⁎10^9^	19.98	10.19
30% D_2_O	80,246	1.387⁎10^9^	19.88	10.19
100% D_2_O	638,424	1.571⁎10^10^	19.61	10.24
^12^C	738,656	4.037⁎10^9^	20.32	10.63
^13^C	234,927	2.154⁎10^9^	20.48	10.64

**Segmentation:** Segmentation of the raw ONT signals was performed using the f5c index and f5c eventalign modules of f5c v1.2 [12], an optimized re-implementation of nanopolish [23]. The eventalign module used the assemblies obtained as a reference sequence. Based on this reference, the bases of each read were mapped to specific segments in the corresponding ONT signal, producing a list of segment coordinates. The f5c index module was executed with the following additional parameters: --scale-events to perform signal normalization, --signal-index to report segment regions, --secondary=no to not allow secondary mappings, --collapse-events to concatenate neighboring segments of the same base, and --pore r9 as source pore.

### 2.4 Data normalization

In nanopore sequencing, reads are represented as a time series of pico-ampere measurements with a resolution of around 3000 Hz. This time series contains both signals from basecalled nucleotides and non-basecalled information from ligated aptamers derived from the sequencing process. As the initial step in data transformation for the prediction model, machine noise typically present in ONT data [26] was removed by a read-level normalization process similar to Z-score calculation.

For each vector of pico-ampere measurements corresponding to the basecalled nucleotides $R_{r}$, the normalized signal $\overset{ˆ}{R_{r}}$ was calculated using scaled median absolute deviation normalization. This normalization method involved calculating the median $\mathrm{med}(R_{r})$ and the median absolute deviation $\mathrm{mad}(R_{r})$ of the vector $R_{r}$.

(1) $${\overset{ˆ}{R}}_{r}=\frac{R_{r}-\mathrm{med}(R_{r})}{\mathrm{mad}(R_{r})}$$

In addition, the non-basecalled signal produced by ligated aptamers, referred to as $X_{r}$, was equally normalized using the same median and median absolute deviation parameters for each read as shown in Eq. (1), yielding the normalized vector ${\overset{ˆ}{X}}_{r}$, the size of which can be of variable length, including zero. This part of the signal was assumed to be free from any isotope influence and was retained to allow investigation of its potential use as a normalization parameter.

### 2.5 Definition and extraction of key statistics

The nanopore signal is commonly assumed to be influenced by a small set of nucleotides, typically 5 nt, passing through the pore at a given time [34]. The data obtained for each read was split into *k*-mers with k=5, representing parts of the read corresponding to 5 nt, based on the segmentation previously obtained. For each read of length *n*, n−4 5-mers were obtained. For every 5-mer of every read, two parameters were extracted from the data: the normalized signal ${\overset{ˆ}{R}}_{r}$ described above, and the time required for the 5-mer to pass through the pore, i.e., the dwell time.

We assumed that 5-mers containing identical sequences would provide highly similar parameters, differing only on the basis of heavy isotope incorporation. The parameters of these identical 5-mers were then combined to generate the 5-mer-based summary statistics. For the normalized signal, two summary statistics were defined: the median across identical 5-mers (signal median), and the median absolute deviation (signal MAD) as the measure for the signal variability between identical 5-mers. The mean and standard deviations of the signal were not selected, as these are more likely to be influenced by outliers in the data. For the dwell time, the median across 5-mers was likewise defined as a summary statistic. This resulted in three 5-mer-based summary statistics: signal median, signal MAD, and dwell time median. As 1024 (i.e., $4^{k}$) different 5-mers exist, the summary statistics were represented by three vectors of 1024 values for each read.

In addition, a read-based summary statistic was calculated: the median of the non-basecalled signal ${\overset{ˆ}{X}}_{r}$ of each read. While ultimately not part of the model, it was assumed that this statistic could be helpful in calibrating the model, as this part of the read data would not contain signatures of heavy isotope incorporation.

Apart from the 5-mer-based summary statistics, the same process was used to collect comparable 3-mer-based and 1-mer-based statistics, with respective lengths of 3⋅64 values and 3⋅4 values per read. While not providing the high resolution of the 5-mer-based statistics, these were used as toy models with lower complexity and computational run times for the analyses discussed below.

### 2.6 Transformation of signal MAD and dwell time median

Both the signal median absolute deviation (MAD) and the dwell time median are represented by strictly positive values. To facilitate downstream modeling, a Box-Cox transformation was applied for both parameters, as shown in Eq. (2).

(2) $$y_{\mathrm{boxcox}(\lambda )}=\left\{ \begin{array}{cc} (y^{\lambda }-1)/\lambda & ,\lambda \neq 0\\ \log y & ,\lambda =0 \end{array}\right.$$

This transformation expanded the domain to include all real numbers, resulting in inputs that exhibit Gaussian-like behavior. The exponent *λ* required for the Box-Cox transformation was determined by fitting the training data using maximum likelihood estimation. The log-likelihood to be maximized was the profile log-likelihood, implemented as shown in Eq. (3).

(3) $$L(\lambda )=(\lambda -1)\sum_{i}^{N}(\log y_{i})-\frac{N}{2}\log(\sum_{i}(b(\lambda )-\bar{b(\lambda )})/N)$$

In this equation, the term $y_{\text{boxcox}}(\lambda )$ is replaced by b(λ) for simplification. For a given *y*, the b(λ) are the Box-Cox transformed values and $\bar{b(\lambda )}$ is their mean. The maximum-likelihood solution was calculated only for the training data, and the resulting *λ* value of 0.40 was subsequently used throughout.

### 2.7 Generalized linear model for isotope detection

Using the *k*-mer-based and read-based summary statistics, a generalized linear model was employed to predict the incorporation of heavy isotopes in DNA molecules on read level (see Eq. (4)). In the model, the vector $m_{r}\in R^{4^{k}}$ holds the signal median from signal regions in ${\overset{ˆ}{R}}_{r}$ corresponding to each *k*-mer of each read *r*. Similarly, $d_{r}\in R^{4^{k}}$ holds the Box-Cox transformed signal MAD. The Box-Cox transformed dwell times for each read and *k*-mer were included as the vector $l_{r}\in R^{4^{k}}$. The scalar $x_{r}$ represents the median of the non-basecalled signal ${\overset{ˆ}{X}}_{r}$ for each read. The constant *α* was used to center the model. During training of the model, the scalar $c_{r}$ encoded the class (with/without heavy isotopes) of the read. When the model was applied, this value represented an error response predicting certainty for membership of a read in a specific class.

(4) $$c_{r}∼{\mathrm{logit}}^{-1}(m_{r}^{T}\cdot \beta +d_{r}^{T}\cdot \gamma +l_{r}^{T}\cdot \delta +x_{r}\nu +\alpha )$$

The parameters β,γ,δ,ν, and *α* are random variables whose distribution was to be determined. Both *ν* and *α* are scalar, while β,γ, and ***δ*** are vector parameters of dimension $4^{k}$. The full Bayesian model is described in the Section 2 of the Appendix. As both the dwell time and the median of the non-basecalled signal showed only negligible impact on the model's ability to correctly predict the modification class (see Appendix Section 2), the final model was simplified as shown in Eq. (5).

(5) $$c_{r}∼{\mathrm{logit}}^{-1}(m_{r}^{T}\cdot \beta +d_{r}^{T}\cdot \gamma +\alpha )$$

In addition, to identify *k*-mers where the impact of heavy isotope incorporation was strong, the resulting vectors were ordered by their absolute values. Individual *k*-mers with high absolute values served as good predictors for isotope incorporation, while *k*-mers with associated absolute values close to zero were not good predictors. The influence of individual nucleotides at specific positions within a *k*-mer was also determined. For a given nucleotide *n* and position *i*, this marginalized importance was calculated as shown in Eq. (6) for a selected vector *p*.

(6) marginal(n,i)=mean{p[kmer[i]=n]}

### 2.8 Training and testing of the model

To train and test the established model, pairwise comparisons between the D_2_O 0% and 100% classes, the D_2_O 0% and 30% classes, and the ^12^C and ^13^C classes were carried out. To ensure the robustness of the model, the read datasets were divided into five subsets of approximately equal size for leave-one-out cross-validation. To address the difference in sizes between datasets of different classes (e.g. D_2_O at 0% and 30%), subsampling was performed on the larger dataset to achieve equal read numbers for each class during training. Four of the subsets were then used for training. Within these subsets, only reads containing all possible *k*-mers were retained. On average, these represented 63% of all reads. The fifth subset was used to test the model's prediction performance. This process was repeated five times, once for each subset for testing. The model response was then averaged over 1 000 posterior predictive samples. The five-fold cross-validation was repeated three times, wherein the five sets were chosen randomly each time, yielding a total of 15 different training sets and corresponding test sets.

### 2.9 Evaluation of the constructed models

To assess the model responses obtained, the number of true positives (TP), i.e., labeled reads assigned to the correct class, true negatives (TN), i.e., unlabeled reads assigned to the correct class, and the corresponding false positives (FP) and false negatives (FN) was determined. From these values, the additional metrics $F_{1}$ score and Matthews correlation coefficient (MCC) were calculated as shown in Eqs. (7) and (8), respectively.

(7) $$F_{1}=\frac{2\cdot TP}{2\cdot TP+FP+FN}$$

(8) $$MCC=\frac{TP\cdot TN-FP\cdot FN}{\sqrt{\left(TP+FP)\cdot (TP+FN)\cdot (TN+FP)\cdot (TN+FN\right)}}$$

Additionally, false discovery rates (FDR) were determined for specific cutoff values of the error response. The FDR represents the proportion of misclassified reads among all reads with error responses that meet the cutoff criteria. The error response was calculated as the absolute (or $L_{1}$) distance to the true class. As there is an increased probability of correct classification for smaller $L_{1}$ distances, reads with an $L_{1}$ distance less than 0.5 to the true class were considered to be correctly predicted.

### 2.10 Implementation details

The model was implemented using PyMC v5.5.0 [1], a Python library that offers a comprehensive framework for probabilistic programming. The complete development environment, including the specific versions of all dependent libraries, is provided as a Nix flake,^1^ ensuring binary reproducibility of the entire project and an exact listing of required dependencies, including those in the transitive closure (the set of all direct and indirect dependencies with their exact versions).

## 3 Results

### 3.1 Sequencing quality and assembly were not impaired by heavy isotope incorporation

The ONT sequencing data obtained from the isotope labeling experiments, using DNA from *Aminobacter* P9b grown on 0%, 30% and 100% D_2_O as well as ^12^C and ^13^C glucose, was first investigated in terms of read quality and output. Consistent PHRED scores of around 20 (indicating 99% accuracy or 1% basecalling error) were obtained for all samples, with no significant differences between samples (see Table 1). Sequencing quality was thus not affected by isotope incorporation. Differences in the number of sequenced reads and basecalled bases followed no discernable pattern with respect to isotope incorporation, with unlabeled samples (0% D_2_O, ^12^C) yielding 102,220 to 738,656 reads and $1.595\times 10^{9}$ to $4.037\times 10^{9}$ bases, and labeled samples yielding 80,246 to 638,424 reads and $1.387\times 10^{9}$ to $1.571\times 10^{10}$ bases. These differences were more likely caused by variations in the amount and structural integrity of input DNA.

Independent assembly of each of the five samples consistently yielded two contigs per sample (see Table 2). The larger contig was approximately 4,973,230 bp in size, likely resembling the bacterial chromosome. It contained essential genes for cellular functions as well as rRNA and tRNA genes. The smaller contig, at 354,980 bp in size, appeared to represent extrachromosomal DNA, containing accessory metabolic genes for methylotrophy and aromatic compound degradation. The length of the respective contigs differed by only a few bases between samples, independent of the isotope labeling.

**Table 2:** **Length of the assemblies.** Shown are the lengths of the longer and shorter contig recovered for each of the five samples.

Assemblies	Chromosome	Plasmid
0% D_2_O	4,937,234 bp	354,994 bp
30% D_2_O	4,937,233 bp	354,986 bp
100% D_2_O	4,937,258 bp	354,984 bp
^12^C	4,937,209 bp	354,974 bp
^13^C	4,937,258 bp	354,993 bp

To exclude any impact on basecalling from isotope incorporation in the labeled DNA, pairwise alignments of the assemblies were constructed. The overall sequence identity for both contigs was above 99.99%. On average, the assemblies differed by 208 indels and 65 mismatches, randomly distributed across the contigs (see Table 3). Thus, on average, only 1 of 22,600 bases differed between the assemblies, demonstrating high consistency and no effect from the isotope incorporation. The original publication for racon [41] reported average identities of 99.90% for *E.coli*, which has a similar genome size, with ≈4.6 million bases.

**Table 3:** **Assembly comparisons and differences for the contigs retrieved.** Shown are the numbers of mismatches (upper triangle) and indels (lower triangle) for each pairwise comparison of the five assemblies, for (**A**) the larger chromosome and (**B**) the smaller plasmid, respectively.

A chromosome	0% D_2_O	30% D_2_O	100% D_2_O	^12^C	^13^C
0% D_2_O		62	65	76	39
30% D_2_O	159		74	76	50
100% D_2_O	190	186		72	54
^12^C	130	162	172		42
^13^C	135	169	181	109	

**B** plasmid	0% D_2_O	30% D_2_O	100% D_2_O	^12^C	^13^C

0% D_2_O		4	3	8	5
30% D_2_O	13		1	6	3
100% D_2_O	14	11		5	2
^12^C	17	14	11		3
^13^C	16	7	12	13	

### 3.2 Nanopore sequencing allows detection of deuterium incorporation

The ability to correctly basecall isotopically labeled DNA suggests that the influence of isotopes on the nanopore signal is negligible compared to that of the nucleotide sequence. To employ this low influence for distinguishing between reads from labeled and unlabeled DNA molecules, *k*-mer-level analysis had to be performed to eliminate the variation caused by the nucleotide sequence. Nanopore data was segmented according to the *k*-mer sizes in our models with *k* = 1, 3, and 5, and separated according to the resulting 4, 64, and 1024 *k*-mers, respectively.

**Fig. 2:**
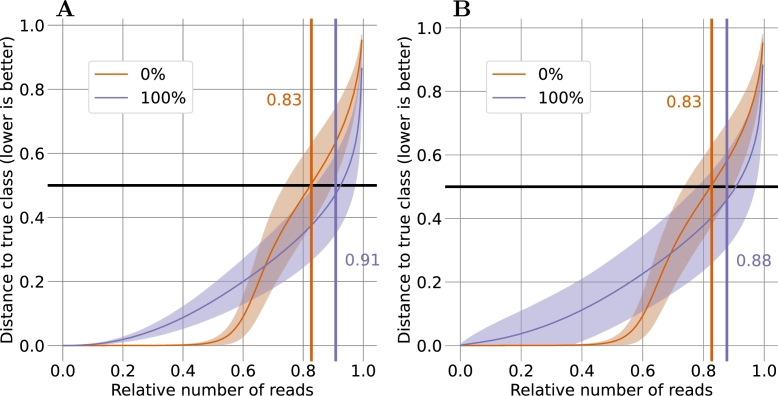
**Response of the model for the 0% and 100% D**_**2**_**O classes.** Shown is the distance to the correct class of each read of (**A**) the training dataset and (**B**) the test dataset, where 0 represents the correct class and 1 the incorrect class. Distances of less than 0.5 indicate correct classification, and the orange and blue numbers in the plots depict the fraction of correct assignments. The x-axis shows the reads in order of increasing distance to the correct class. To account for differences in read numbers between classes, the axis is normalized to values between 0 and 1. Reads from the 0% D_2_O class are shown in orange and reads from the 100% D_2_O class are shown in blue. Lines represent the mean and shaded areas show one standard deviation based on the triplicate five-fold cross-validation data.

We first constructed a model to differentiate between the 0% D_2_O and 100% D_2_O classes. The selected reads were split into five sets (see Section 2.8) for cross-validation. Per class, the training set contained between 77,270 and 82,604 reads and the test sets contained between 18,379 and 23,713 reads. Initially, we employed the “No U-turn sampler” (nuts) [13], which yielded promising fits for the 1-mer and 3-mer models (given the simplicity of these models), with a correct classification rate of 0.49 and 0.65 for the 0% D_2_O class and 0.66 and 0.84 for the 100% class, respectively (see Table 4). However, at more than 100 hours, the required computational time predicted for the 5-mer model was prohibitively long. To address this issue, we turned to variational inference (see Appendix Section 2 and [7]) in conjunction with the “adagrad” optimizer [11]. The resulting model fits for the 1-mer and 3-mer models were comparable to those of NUTS, while running time, even for the 5-mer model, was reduced to just a little more than 30 minutes (see Table 4). The final 5-mer model resulted in an average correct classification rate of 0.86 for the 0% D_2_O class and 0.88 for the 100% D_2_O class (see Fig. 2).

**Table 4:** **Behavior of the isotope classification model for D**_**2**_**O under differing conditions.** The model was trained using two distinct algorithms, namely nuts and adagrad with variational inference (see Section 3.2) and three different *k*-mer sizes (1, 3, 5). The table displays total running times for fitting the models, excluding the initial data preparation phase (see Appendix Section 1). The train and test columns provide the true positives (TP) and true negatives (TN) under differing conditions as measures of classification performance. For the most informative case of testing with 5-mers, the metrics *F*_1_, MCC, and precision are additionally provided. Bold values represent averages over the five-fold cross-validation runs. For the 30% regime, the 1-mer model was omitted. All calculations were performed on an AMD Ryzen 9 3900X with 64 GB RAM.

			TN	TP	TN	TP	F_1_	MCC	Prec
			Train		Test				
Model	*k*	Time	0%	100%	0%	100%			

nuts	1	10 m:55 s	0.49	0.66	0.47	0.68			
	3	>3*h*	0.65	0.84	0.63	0.75			
	5	>100*h*	–	–	–	–	–	–	–

adagrad	1	1 m:01 s	0.50	0.66	0.48	0.68			
	3	3 m:01 s	0.60	0.86	0.56	0.86			
	**5**	**10 m:51 s**	**0.83**	**0.91**	**0.83**	**0.88**	**0.86**	**0.71**	**0.84**

			Train		Test				
Model	*k*	Time	0%	30%	0%	30%			

adagrad	3	1 m:32 s	0.67	0.68	0.65	0.69			
	**5**	**15 m:11 s**	**0.87**	**0.88**	**0.84**	**0.87**	**0.86**	**0.71**	**0.84**

More than six out of seven reads were thus correctly classified. Validation of the established model with the test dataset resulted in a highly similar success rate and error response curves, with correct classification rates of 0.86 and 0.88 for the 0% and 100% D_2_O classes, respectively. This indicates that the incorporation of deuterium leads to detectable changes in nanopore signals.

**Fig. 3:**
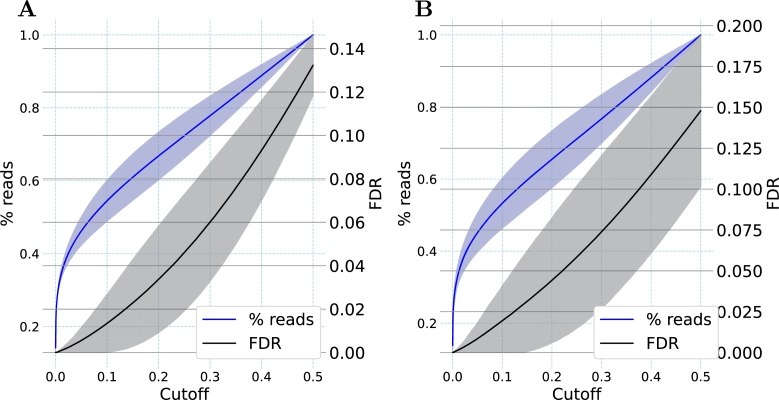
**Impact of error response cutoffs for the 0% and 100% D**_**2**_**O classes.** Shown are the false discovery rate (FDR, black) and the fraction of reads (blue) meeting the cutoff for (**A**) the training dataset and (**B**) the test dataset. The lines represent the mean and the shaded areas represent one standard deviation based on the triplicate five-fold cross-validation. Cutoffs indicate the maximal distance from the extreme logistic regression values of 0.0 and 1.0. A cutoff of, e.g., 0.05, includes only those reads with values either ≤0.05 or ≥0.95.

To provide a quantitative value for the reliability of the classifications provided by this model, we determined the false discovery rates (FDR) relative to different cutoff values for the error response (see Fig. 3). While a low false discovery rate is desirable due to the resulting low number of misclassified reads, the disadvantage of this is that more reads will remain unclassified because their error response is above the cutoff. We found that for an FDR below 0.05 (i.e. less than 5% misclassifications), a cutoff value of 0.30 was required for the test dataset. With this cutoff, 75.2% of the reads could still be classified. When a stricter FDR below 0.01 (i.e. less than 1% misclassifications) was used, a cutoff value of 0.11 was required, still allowing for the classification of 53.5% of the reads in the test dataset.

### 3.3 Established model allows robust prediction also for labeling with 30% D_2_O

**Fig. 4:**
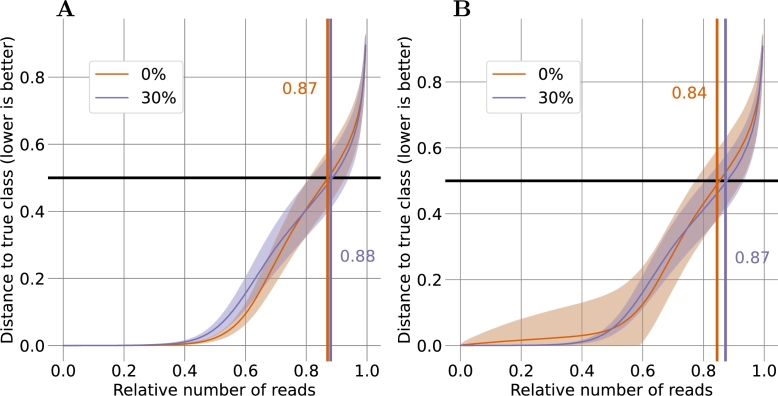
**Response of the model for the 0% and 30% D**_**2**_**O classes.** Shown is the distance to the correct class of each read of (**A**) the training dataset and (**B**) the test dataset, where 0 represents the correct class and 1 the incorrect class. Distances of less than 0.5 indicate correct classification, and orange and blue numbers in the plots depict the fraction of correct assignments. The x-axis shows the reads in order of increasing distance to the correct class. To account for differences in read numbers between classes, the axis is normalized to values between 0 and 1. Reads from the 0% D_2_O class are shown in orange and reads from the 30% D_2_O class are shown in blue. Lines represent the mean and shaded areas show one standard deviation based on the triplicate five-fold cross-validation data.

Based on the established model for the distinction of reads from the 0% D_2_O and the 100% D_2_O class, we next attempted to achieve a separation of the 0% D_2_O and 30% D_2_O classes. As high concentrations of D_2_O have been reported to inhibit microbial growth [6], employing 30% D_2_O is a more reasonable alternative for metabolic labeling experiments. Applying the “adagrad”-based model to training sets with 61,355 to 66,496 reads and test sets with 14,350 to 19,491 reads provided an acceptable correct classification rates at sub-hour training times (see Table 4). The five-fold cross-validation yielded correct classification rates of 0.88 for the 0% D_2_O class and 0.86 for the 30% class in the test dataset (see Fig. 4), i.e., fewer than one out of seven reads were incorrectly classified. Further, as indicated by the initially flat error response curves in Fig. 4, the majority of reads for both classes were classified correctly with very high confidence (an error response <0.1).

**Fig. 5:**
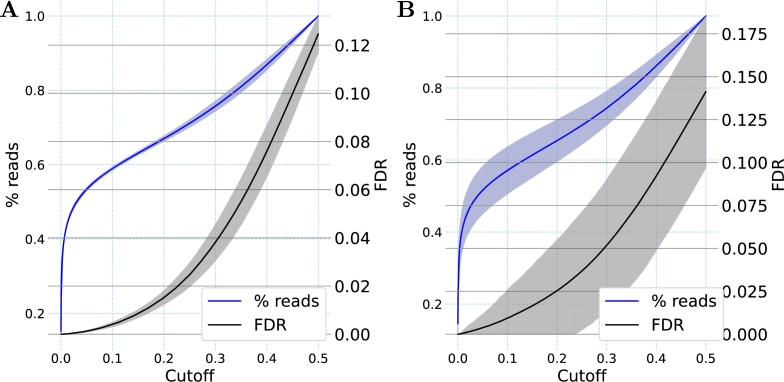
**Impact of error response cutoffs for the 0% and 30% D**_**2**_**O classes.** Shown are the false discovery rate (FDR, black) and the fraction of reads meeting the cutoff (blue) for (**A**) the training dataset and (**B**) the test dataset. The Lines represent the mean and the shaded areas represent one standard deviation based on the triplicate five-fold cross-validation. Cutoffs indicate the maximal distance from the extreme logistic regression values of 0.0 and 1.0. A cutoff of e.g. 0.05 includes only those reads with values either ≤0.05 or ≥0.95.

As before, FDRs related to different error response cutoffs were determined for the 0% D_2_O and 30% D_2_O class comparison (see Fig. 5). For an FDR below 0.05 in the test set, a cutoff value of 0.34 was required, allowing the classification of nearly 80% of the reads. To reach the stricter FDR of 0.01, a cutoff value of 0.17 was necessary. With this cutoff, more than 64% of all reads in the test dataset still reached the cutoff and were classified. This shows that despite the lower incorporation of heavy isotopes with 30% D_2_O, as compared to 100% D_2_O, a robust differentiation of these two classes was achieved for a substantial proportion of the reads.

### 3.4 No reliable detection of ^13^C signal

**Fig. 6:**
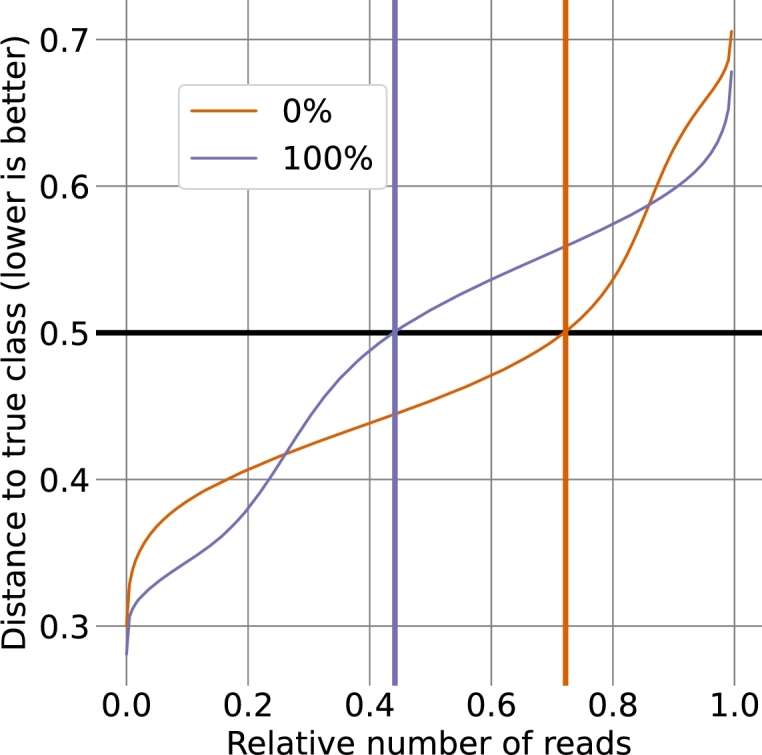
**Response of the model for the**^12^**C (0%) and**^13^**C (100%) class.** Shown is the distance to the correct class of each read of the training dataset, where 0 represents the correct class and 1 the incorrect class. Distances around 0.5 indicate a low certainty of the classification. The x-axis shows the reads in order of increasing distance to the correct class. To account for differences in read numbers between classes, the axis is normalized to values between 0 and 1. Reads from the ^12^C class are shown in orange and reads from the ^13^C class are shown in blue. Data is based on 129,478 reads in each class.

Following the successful classification of deuterium labeled and unlabeled reads, we applied the same approach to enable a separation of the ^12^C (unlabeled) and ^13^C classes. Using the “adagrad”-based approach, initial training of a 1-mer and a 3-mer model failed to lead to promising results. Nevertheless, a 5-mer model was trained with 129,478 reads for each class. This model achieved an error response of only 0.73 for the ^12^C class and 0.45 for the ^13^C class. In contrast to the model employing 30% D_2_O labeling, the error response curves showed no initial flat section representing highly certain classifications (see Fig. 6**A**) Instead, the curves resembled the underlying logit function, suggesting a mostly random assignment of the reads to the two classes.

### 3.5 Effect of deuterium incorporation differs by *k*-mer

**Fig. 7:**
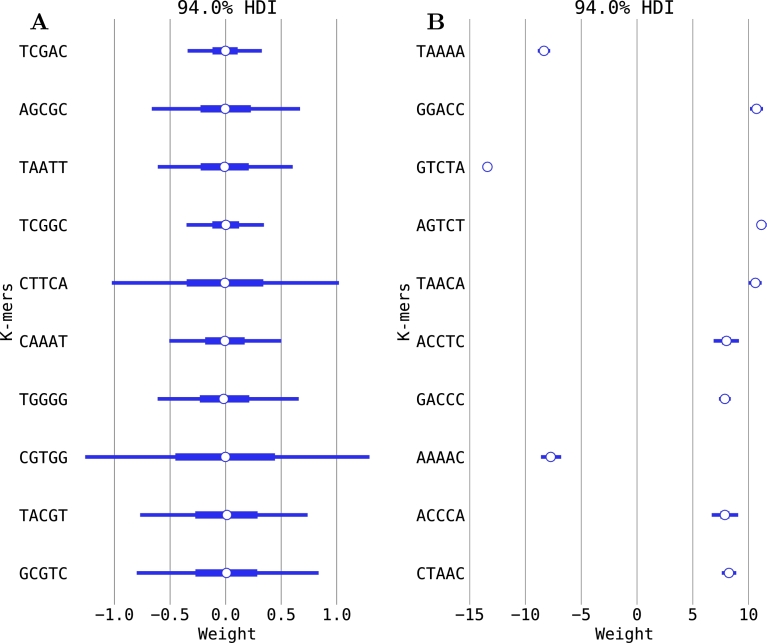
**Influence of isotope incorporation on the median parameter of the nanopore signal for different 5-mers.** Shown are (**A**) the ten 5-mers with the lowest absolute weights and (**B**) the ten 5-mers with the largest absolute weights of the 0% vs. 30% D_2_O comparison. Central points indicate the posterior mean of the parameter, boxes the central quartiles, and thin lines the 94% highest posterior density interval (HDI) likely to be significant. 5-mers in bold likewise show the strongest influence on signal MAD. Average data obtained from five-fold cross-validation is shown. Note the differing scales for the left and right plots. The *k*-mers and their values were obtained by ordering the median vector acquired from Eq. (5), as described in Section 2.7.

**Fig. 8:**
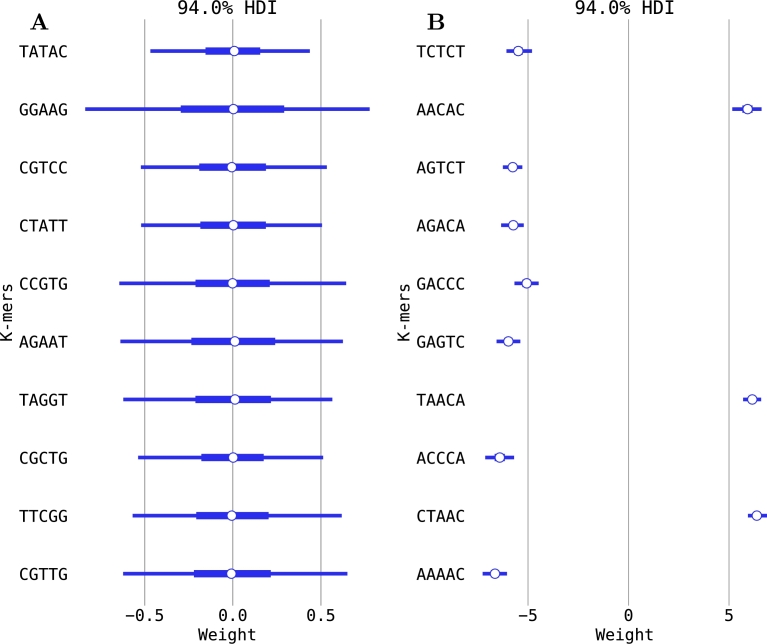
**Influence of isotope incorporation on the signal MAD parameter of the nanopore signal for different 5-mers.** Shown are (**A**) the ten 5-mers with the lowest absolute weights and (**B**) the ten 5-mers with the largest absolute weights of the 0% vs. 30% D_2_O comparison. Central points indicate the posterior mean of the parameter, boxes the central quartiles, and thin lines the 94% highest posterior density interval (HDI). 5-mers in bold likewise show the strongest influence on median signal. Average data obtained from five-fold cross-validation is shown. Note the differing scales for the left and right plots. The *k*-mers and their values were obtained by ordering the median absolute deviation vector acquired from Eq. (5), as described in Section 2.7.

Because the model for the classification of isotope-labeled and unlabeled reads is based on signal variations for each *k*-mer, we determined whether the responses of the different *k*-mers to isotope incorporation differed. For each *k*-mer of the 5-mer model, both the signal median and the signal MAD were compared between the 0% D_2_O and the 30% D_2_O classes. The *k*-mer identity exhibited a strong influence on both values, with some *k*-mers exhibiting no difference between the two classes, while others were strongly affected, thus also strongly influencing the model (see Fig. 7, Fig. 8, Appendix Fig. 2).

For the signal median, the majority of the strongly influenced *k*-mers exhibited a higher signal in the labeled than in the unlabeled class. The signal MAD, however, was lower in the labeled class for most of the strongly influenced *k*-mers. Those *k*-mers with the strongest influence on the signal median differed from those with the strongest influence on signal MAD. In addition, no pattern was observed between *k*-mer identity and the strength of the effect of the isotope incorporation. The presence of specific nucleotides or motifs in the *k*-mer did not appear to have any systematic effect.

### 3.6 Nucleotides reveal only low positional importance

**Fig. 9:**
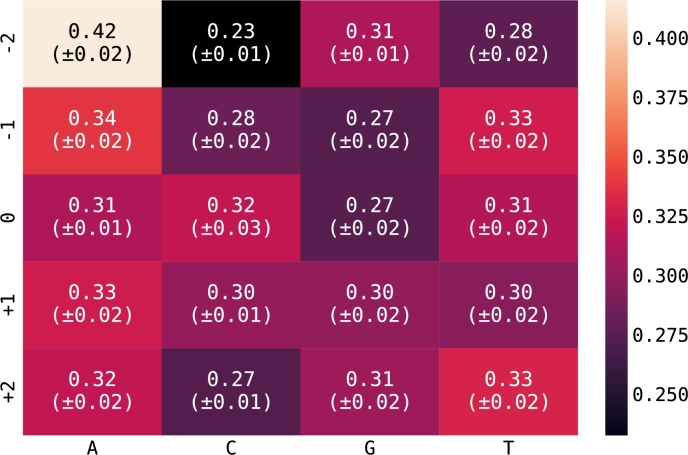
**Marginalized importance of nucleotides at each position in a 5-mer.** Data was derived from the model comparing 0% and 30% D_2_O. Brighter colors and higher numbers indicate a higher impact of the respective nucleotide on the classification model, while darker colors and lower numbers indicate a lower impact. The statistical behavior of nucleotides A and C at an extreme position (-2) is exceptional and reflected by the large number of A nucleotides (4 and 5 out of 10, respectively) at position (-2) in the top-most 5-mers with high weights, as seen in Fig. 7, Fig. 8. Eq. (6) was used to calculate these values.

As the next step, we tested whether the relative position of a given nucleotide in the *k*-mer influences the relative importance of the respective *k*-mer for the 0% vs. 30% D_2_O model. In general, the statistical influence of all positions and nucleotides was similar (see Fig. 9). The strongest influences were observed for adenosines, with a remarkable uptick in importance at the ‘-2’ position. Signals of *k*-mers with this nucleotide thus appeared to be more strongly affected by deuterium incorporation. Higher variations across nucleotides were, in general, also observed in the ‘-2’ position, leading to both the negative and positive extremes observed. The relative importance at the ‘0’, ‘+1’, and ‘+2’ positions was much more uniform across nucleotides. It has been suggested that the signal of a *k*-mer is particularly influenced by the ‘-1’, ‘0’, and ‘+1’ positions of a 5-mer, representing its center [34]. Our results indicate that the outer positions as well, especially ‘-2’, may have a stronger influence on the observable change in nanopore signal due to deuterium inclusion. Neither the lack of pattern for the observed differences between *k*-mers nor the low positional correlation of the nucleotide importance pointed to any mechanistic explanation of the effect of deuterium incorporation on the nanopore signal. However, as our classification models take all *k*-mers into account, and *k*-mers with a greater differences between unlabeled and labeled reads have a higher influence on the model, successful classification was possible, independent of mechanistic insights.

### 3.7 Further parameters not affecting class prediction

Along with signal median and signal MAD, we investigated whether additional information could improve the predictive capabilities of the model. The dwell time, defined as the number of signal points recorded for a nucleotide, was included analogously to median and MAD for each 5-mer. This inclusion did not significantly impact the predictive power of our model (see supplemental Fig. 3). Surprisingly, however, it led to an increase in variance among cross-validation runs. Therefore, dwell times were not incorporated into the main model presented in this study.

Further, along with the main sequence, information contained in the non-basecalled signal $x_{r}$ of each read was considered. An expanded model was derived to explore the potential “de-biasing” effect of this information on the normalized signal. However, this additional information did not significantly influence or enhance the performance of the algorithm. The probability mass of the “pre-scale” parameter, which represents the influence of the non-basecalled signal, was found to be concentrated in the range of 0 to 0.05, effectively reducing its impact to zero (see Appendix Fig. 1).

## 4 Discussion

Nanopore sequencing has revolutionized genomics and molecular biology by providing a means to acquire data from individual DNA and RNA molecules, facilitating an untargeted approach for detecting nucleic acid modifications. In the study presented, we aimed to determine whether ONT sequencing would enable the detection of heavy isotope incorporation. Our results revealed the clearly detectable impact of deuterium incorporation on the nanopore signal, supporting our hypothesis for this isotope. The model developed allows a robust distinction between deuterium-labeled and unlabeled DNA molecules, even when deuterium incorporation is only partial, as in the case of metabolic labeling with 30% D_2_O. In contrast, no differentiation of ^13^C-labeled and unlabeled DNA based on the nanopore signal was possible, refuting our hypothesis with respect to ^13^C. These observations are in line with the commonly described biological effects of isotope labeling, which manifest in frequently observed inhibitions of bacterial growth at higher D_2_O concentrations [6], [31], while for ^13^C, such effects have generally not been reported, even for fully labeled substrates. Hydrogen occurs at the surface of biomolecules, and when exchanged with deuterium, the atomic weight doubles, possibly leading to a strong kinetic isotope effect [43]. Carbon, in contrast, forms the backbone of biomolecules, and atomic masses increase by only 1/12th with heavy isotope incorporation. This could lead to a stronger impact of the incorporation of deuterium on the function of biomolecules, which is often based on molecular recognition. As the nanopore is a biomolecular structure, the same effects that inhibit bacterial growth may also influence the pico-ampere signal recorded during sequencing.

From a statistical perspective, our model has a number of desirable features. As a generalized linear model, each parameter and its influence on the model can be readily explained. In addition, “trial runs” with a reduced *k*-mer width can be used to perform initial validity checks. The advantage, in particular of the 3-mer model, is that running times are short enough to allow for rapid testing and prototyping. In addition, the 3-mer model has already demonstrated highly predictive performance and may possibly already be able to be successfully employed. This smaller model, and, to a lesser extent, the 1-mer model, also provide evidence that there was no accidental overfitting of the model. In addition, the use of variational inference [7] retains most of the predictive power of the fully Bayesian model at a fraction of the running time costs. The running times of the model are thus distinctly shorter than those needed for training a deep-learning model. The only major cost in running time was, in fact, for the preprocessing steps used to collect summary statistics for each read. However, these are only required to be collected once and can be run in parallel on each Oxford Nanopore file, of which there are typically several hundred. Finally, the approach introduced here could potentially also prove useful for tracking modifications other than isotope incorporation in DNA molecules.

The model presented provides predictions on a per-read level, and as such data preparation was geared towards efficient calculation of summary statistics for this level. This is in contrast to other Oxford Nanopore applications that require nucleotide-level resolution to pinpoint nucleotide-specific modifications [34], [3], [9]. However, this model would be suitable for potential applications in the area of microbial ecology, where the goal is to collect information on the level of organisms. The predictions generated by our model allowed an accurate distinction between the unlabeled and labeled classes for 85% of the sequenced reads. When sequencing an unknown mixture of labeled and unlabeled DNA molecules, e.g., following D_2_O labeling in a complex microbial community, the methods outlined could be used to apply FDR cutoffs of 0.01 or 0.05 to exclude reads with uncertain classifications. Similar methods to control the overall validity of large data sets are also used for other omics approaches, such as metaproteomics [35], [42]. Our results indicate that the degree of certainty in predictions for the 0% and 30% D_2_O classes is generally high enough to ensure that a substantial proportion of the reads would be retained after this step. To further increase prediction certainty on the organism level, sequencing reads can be subjected to metagenomic binning, to group reads and assign them to their organism of origin [32], [4], [33]. Because it forms a consensus of read-level predictions for a metagenomic bin, this approach promises a high level of certainty with regard to the distinction of organisms with and without D_2_O incorporation.

The potential for detecting the incorporation of heavy isotopes from metabolic labeling in DNA molecules provides a promising starting point for the future development of new methods for tracking active microbes. A direct link between incorporation, representing activity, and identity, derived from the obtained sequence information, would significantly increase the informative value of DNA-SIP experiments. Techniques providing similar links have been introduced for other SIP methods as well. In Metaproteome-SIP, for example, the same mass spectral signals serve for both protein identification and quantification of the heavy isotope incorporation [5], [36]. For nucleic acid-based SIP approaches, however, no detection method is yet available for achieving this combination, which represents a severe limitation. Compared to other SIP approaches, nucleic acid based methods can provide high throughput, de novo information about the organisms under investigation, hence overcoming this limitation is highly desirable. Leveraging this great advantage would enable research into activity of previously unclassified and unexplored microbial groups, the microbial dark matter [14], [37], whose functions are currently one of the most interesting areas of research in microbial ecology. Further, the detection of heavy isotope incorporation at the single molecule level potentially opens the door to further developments that would allow insight into differences in activity within otherwise genetically identical microbial populations. These advances have the potential to provide new mechanistic information on the dynamics of microbial growth and the parameters that impact metabolic activity.

## Funding

This study is funded by the 10.13039/501100001659Deutsche Forschungsgemeinschaft (DFG, German Research Foundation) under Germany's Excellence Strategy – EXC 2051 – Project-ID 390713860 and the Collaborative Research Centre AquaDiva of the Friedrich Schiller University Jena Project-ID 218627073 – SFB 1076. This work was funded, in part, by the German state of Thuringia via the 10.13039/501100004403Thüringer Aufbaubank (2021 FGI 0009), the 10.13039/100007569Carl-Zeiss-Stiftung within the program Scientific Breakthroughs in Artificial Intelligence (Project “Interactive Inference”), and the DFG NFDI 28/1 “NFDI4Microbiota” (Project-ID 460129525).

## CRediT authorship contribution statement

**Christian Höner zu Siederdissen:** Writing – original draft, Software, Methodology, Formal analysis, Conceptualization. **Jannes Spangenberg:** Writing – original draft, Software, Data curation. **Kevin Bisdorf:** Resources, Investigation. **Sebastian Krautwurst:** Resources, Data curation. **Akash Srivastava:** Resources. **Manja Marz:** Writing – original draft, Funding acquisition, Conceptualization. **Martin Taubert:** Writing – original draft, Methodology, Funding acquisition, Formal analysis, Conceptualization.

## Declaration of Competing Interest

The authors declare no conflict of interest.

## Appendix A Supplementary material

MMC Model details and supplementary figures. MMC

## Footnotes

1 https://github.com/choener/Prj-IsotopeSeparation.
