# Supplementary material for: Nanopore sequencing enables novel detection of deuterium incorporation in DNA
Source: Comput Struct Biotechnol J. 2024 Oct 3;23:3584–94. doi: 10.1016/j.csbj.2024.09.027 (PMC11832021; doi:10.1016/j.csbj.2024.09.027)
Supplement: MMC — Model details and supplementary figures. [file mmc1.pdf]

Nanopore sequencing enables novel detection of deuterium incorporation in DNA

Christian Höner zu Siederdissen[1,c], Jannes Spangenberg[1], Kevin Bisdorf[1], Sebastian Krautwurst[1], Akash Srivastava[1], Manja Marz[1,4,5,6], and Martin Taubert[2,3,c]

[1]RNA Bioinformatics and High-Throughput Analysis, Friedrich Schiller University Jena, Jena, Germany
[2]Aquatic Geomicrobiology, Institute of Biodiversity, Faculty of Biological Sciences, Friedrich Schiller University Jena, Dornburger Str. 159, 07743 Jena, Germany
[3]Balance of the Microverse, Cluster of Excellence, Friedrich-Schiller-University Jena, Grüne Aue, 07745 Jena, Germany
[4]European Virus Bioinformatics Center, Jena, Germany
[5]German Center for Integrative Biodiversity Research (iDiv) Halle-Jena-Leipzig, Leipzig, Germany
[6]FLI Leibniz Institute for Age Research, Jena, Germany
[c]corresponding authors

September 23, 2024

1 Generation of summary statistics

The Oxford Nanopore basecalling software groups reads into sets of $4,000$ reads each into files. We associate two files of summary statistics with each read file. These files contain the statistics required for the generalized linear model and have to be created only once. We require segmented ONT data. This makes it possible to calculate the median and median absolute deviation of the signal for each nucleotide of each read. In addition, we collect the dwell time, i.e. the number of signal points associated with a nucleotide.

Furthermore, the median and median absolute deviation over the signal data for the whole read (only including actual read information, excluding poly-A, barcode, etc) are extracted to allow for normalization of the read data.

The per-nucleotide median and median-absolute deviation data are stored both, in raw pico-ampere form, as well as already normalized based on the

whole-read information.

2 Generalized linear model

We propose a fully Bayesian generalized linear model to distinguish between reads containing different levels of isotopes. While the median data is either easily normalized (see Main Sec. 3.4) or stored normalized (see Sec. 1) and then directly usable, other parameters are positive-only and are transformed using the Box-Cox transform (Main Sec. 3.6) first. Transformed parameters include the median absolute deviation and dwell times. The Box-Cox transform can be inverted, given the λ parameter, but its main use is that it further simplifies downstream modelling as the transformation symmetrizes the data and allows for unconstrained priors.

In addition, for each read we also have a scalar relating the median of the per-read signal part to the per-read "non-signal" part (containing poly-A and barcode information, for example). Finally, for training purposes we know the isotope label of each read (e.g. 0% D_2O vs. 30% D_2O).

As described in Main Sec. 3.4 we consider $k \in \{1, 3, 5\}$-mers, where $k = 1$ and $k = 3$ are simplified "toy" models that can be trained much faster but are likely have less predictive power.

For each read $r \in [1, \ldots, N]$, the summary statistics just mentioned are available. The following indepedent variables are inputs to the model. The vectors $\boldsymbol{m}_r$ of median signal responses, together with $\boldsymbol{d}_r$ of corresponding transformed median absolute deviations, and the transformed dwell time $\boldsymbol{l}_r$ are each k-mer specific and of dimension 4^k. The median prefix signal x_r is scalar.

Finally, α is the intercept.

$$
\begin{aligned}
\omega &\sim \mathcal{B}(0.5, 0.5) \\
\beta &\sim \mathcal{N}(0^{4^k}, \sigma = (1, \ldots, 1)^T) \\
\gamma &\sim \mathcal{N}(0^{4^k}, \sigma = (1, \ldots, 1)^T) \\
\delta &\sim \mathcal{N}(0^{4^k}, \sigma = (1, \ldots, 1)^T) \\
\nu &\sim \mathcal{N}(0, \sigma = 1) \\
p_r &\sim \text{logit}^{-1}((\boldsymbol{m}_r - \omega \cdot x_r)^T \cdot \boldsymbol{\beta} + \boldsymbol{d}_r^T \cdot \boldsymbol{\gamma} + \boldsymbol{l}_r^T \cdot \boldsymbol{\delta} + x_r \nu + \alpha) \\
c_r &\sim \text{Bn}(p)
\end{aligned}
\tag{1}
$$

We write $P(\alpha, \boldsymbol{\beta}, \boldsymbol{\gamma}, \boldsymbol{\delta}, \nu, \omega | \boldsymbol{m}, \boldsymbol{d}, \boldsymbol{\delta}, x, k)$ and make explicit the role of k-mer length.

The fully Bayesian model described above has prohibitely long execution times as shown in Main Table 4 even using the *nuts* sampler [2]. We instead use variational inference [1] to approximate the probability distribution in Eqn. 1. Variatianal inference proceeeds by finding a simpler distribution that is close to the original distribution, and then optimizes its parameters to minimize their

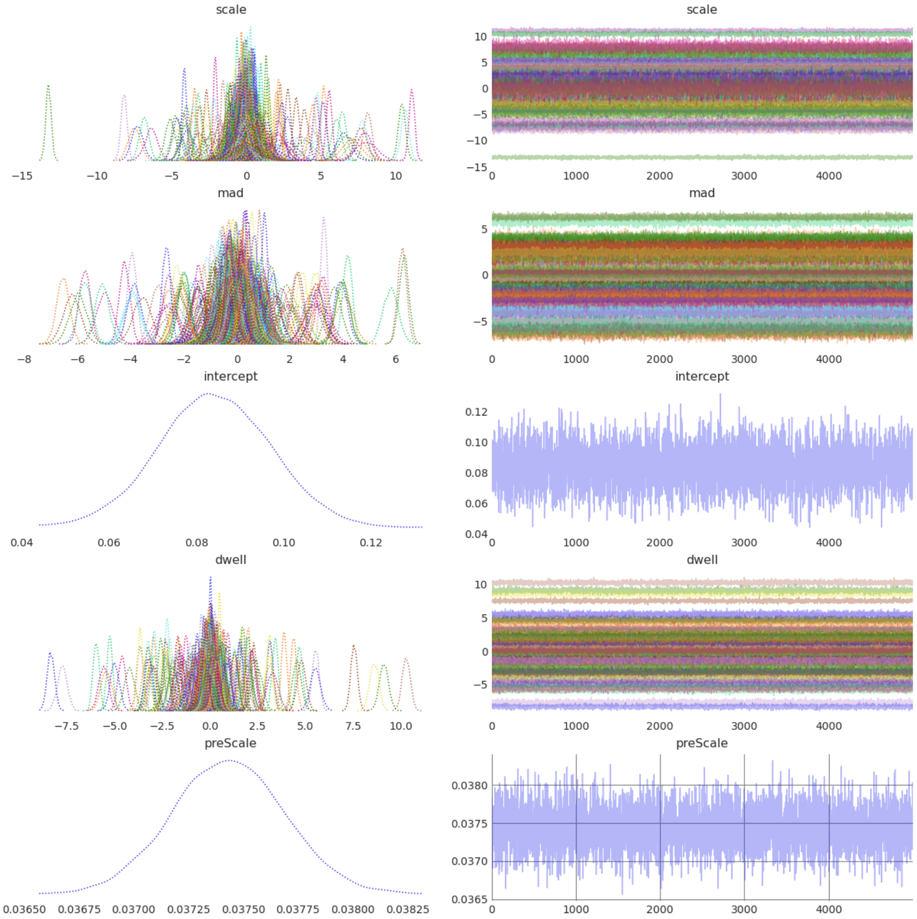

Figure 1: The trace from the adagrad sampling process for the isotope model. One can identify a small set of scale and mad parameters that strongly influence the model response. The pre-scale parameter is estimated to be approximately 0.0, which indicates that signal information from the adapter and poly-A can not be used to improve the normalization of the data.

difference. As a result, we have an efficient algorithm that scales well with both, the k-mer length and the number of data points.

Fig. 1 provides a trace of the execution of this model. Note in particular the small set of parameters that are relevant, and the strong evidence for the "prescaling" parameter to be zero.

3 Detailed analysis of the most influential parameters during cross-validation

The selection of the most influential k-mers, i.e. those that most strongly influenced by the presence of D_2O needs to be stable across experiments for correct prediction of D_2O presence. For each cross-validation run, we selected the ten most significant 5-mers, as well as the three least significant ones. A priori, we expect significant overlap between the most significant 5-mers, while the ordering of the least significant ones will be essentially random.

Forming the union of the ten most significant 5-mers across five cross-validation runs yields thirteen 5-mers. These are depicted together with their individual parameter mean and standard deviations in Fig. 2[A]. Note the strong consistency between all sets of five measurements each, which provides strong evidence that selection of influential parameters for the scale parameter is very stable.

For insignificant parameters, we selected the three least influential ones which gives us a total of fifteen 5-mers across all runs, cf. Fig. 2[B]. This behaviour is expected, but notice how again all means and standard deviations are in agreement.

4 Inclusion of dwell times

Our main model, described in Main Sec.3.7, Main Eqn. 4 excludes dwell times, even though they are part of the fully Bayesian model shown in Sec. 2, Eqn.1. Main Fig. 3 depicts the reason for this choice. Both variants have very close mean responses, but the inclusion of dwell times (Main Fig. 3[C,D]) leads to higher variance within the error response compared to the model without dwell times (Main Fig. 3[A,B]).

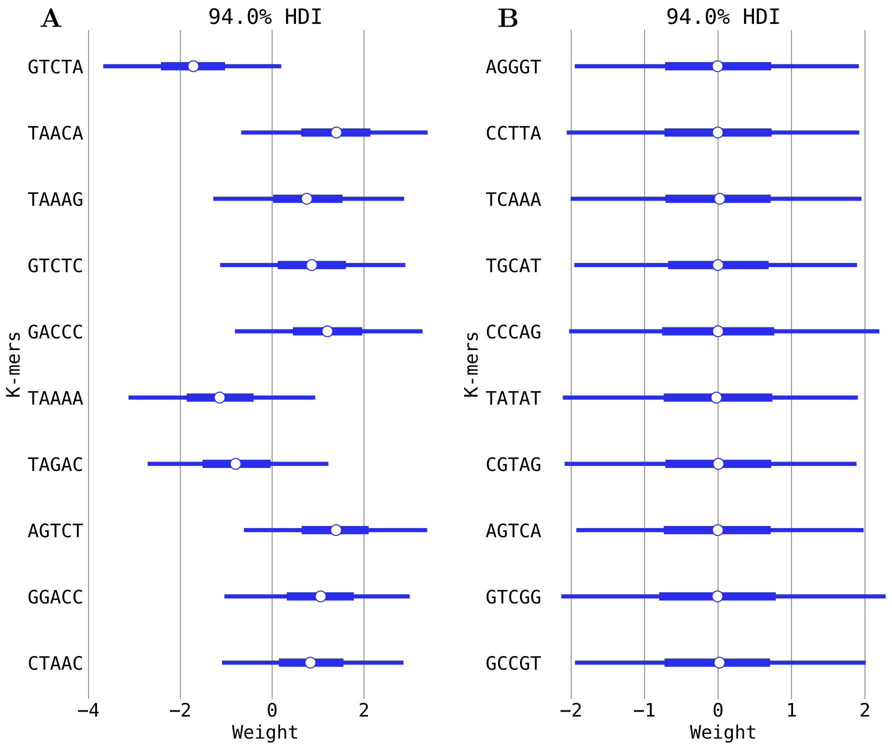

Figure 2: **Comparative analysis of influence of isotope incorporation on the median parameter between the subsets used for cross-validation.** In each of the five cross-validation runs of the 0% vs. 30% D_2O comparison, the ten most significant (**A**) and three least significant (**B**) 5-mers for the scaling variable were identified, resulting in a set of 13 and 15 5-mers, respectively. Coloring specifies the respective cross-validation runs, central points indicate the posterior mean of the parameter, boxes the central quartiles, and thin lines the 94% highest posterior density interval (HDI) likely to be significant. Results indicate that 5-mer importance is consistent across cross-validation runs.

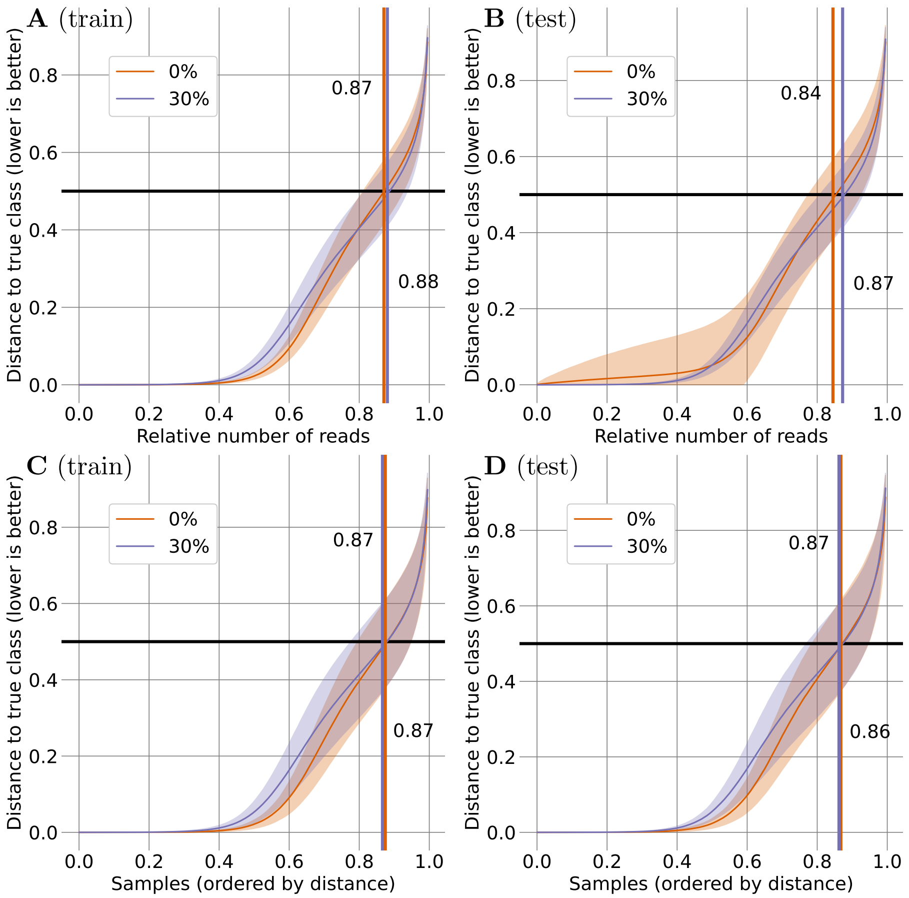

Figure 3: Comparison of the error response in the absence (**A**,**B**) and presence (**C**,**D**) of dwell times as part of the model is presented. Subfigures **A**,**C** depict training data, while subfigures **B**,**D** depict test data. The mean and standard deviation of five-fold cross-validation test results are displayed for all cases. The relative number of correctly predicted reads is nearly identical, differing by approximately 1.5%. However, the inclusion of dwell times leads to an increase in variance.
